# *trans*-Dichloridobis{dicyclo­hex­yl[4-(dimethyl­amino)­phen­yl]phosphane-κ*P*}platinum(II) dichloro­methane disolvate

**DOI:** 10.1107/S1600536812048465

**Published:** 2012-11-30

**Authors:** Wade L. Davis, Reinout Meijboom

**Affiliations:** a Research Centre for Synthesis and Catalysis, Department of Chemistry, University of Johannesburg (APK Campus), PO Box 524, Auckland Park, Johannesburg, 2006, South Africa

## Abstract

In the title complex, *trans*-[PtCl_2_{P(C_6_H_11_)_2_(4-Me_2_NC_6_H_4_)}_2_]·2CH_2_Cl_2_, the Pt^II^ atom is located on an inversion centre, resulting in a *trans*-square-planar geometry. Important geometric parameters are the Pt—P and Pt—Cl bond lengths of 2.3258 (6) and 2.3106 (6) Å, respectively, and the P—Pt—Cl angles of 89.64 (2) and 90.36 (2)°. The effective cone angle for the dicyclo­hex­yl[4-(dimethyl­amino)­phen­yl]phosphane unit was calculated to be 164°. The compound crystallizes with two dichloro­methane solvent mol­ecules; one of which is severely disordered and was treated using the SQUEEZE routine in *PLATON* [Spek (2009 ▶). *Acta Cryst.* D**65**, 148–155].

## Related literature

For a review on related compounds, see: Spessard & Miessler (1996 ▶). For related compounds, see: Johansson *et al.* (2002 ▶). For similar *R*-P_2_PtCl_2_ compounds, see: Lutz *et al.* (2005 ▶). For the synthesis of starting materials, see: Drew & Doyle (1990 ▶). For use of the SQUEEZE routine in *PLATON* to remove the contribution of disordered solvents, see: Spek (2009 ▶). For background to cone angles, see: Tolman (1977 ▶); Otto (2001 ▶).
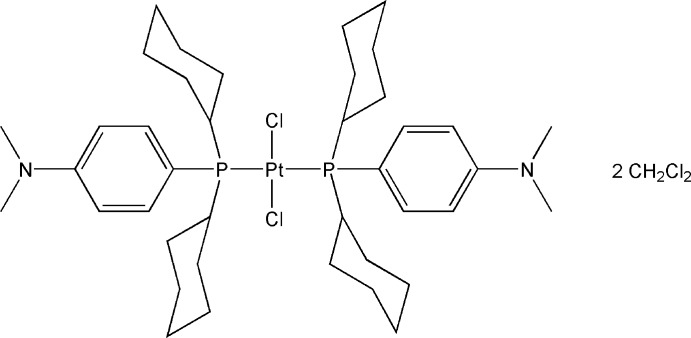


## Experimental

### 

#### Crystal data

[PtCl_2_(C_20_H_32_NP)_2_]·2CH_2_Cl_2_
*M* *_r_* = 1070.70
Monoclinic, 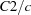

*a* = 19.4146 (9) Å
*b* = 13.1517 (6) Å
*c* = 19.3459 (9) Å
β = 94.660 (2)°
*V* = 4923.4 (4) Å^3^
*Z* = 4
Cu *K*α radiation
μ = 9.16 mm^−1^
*T* = 100 K
0.26 × 0.24 × 0.16 mm

#### Data collection

Bruker APEX DUO 4K CCD diffractometer
Absorption correction: multi-scan (*SADABS*; Bruker, 2008 ▶) *T*_min_ = 0.199, *T*_max_ = 0.322
56178 measured reflections
4239 independent reflections
4069 reflections with *I* > 2σ(*I*)
*R*_int_ = 0.045

#### Refinement

*R*[*F* ^2^ > 2σ(*F* ^2^)] = 0.025
*wR*(*F* ^2^) = 0.067
*S* = 1.08
4239 reflections
244 parameters
H-atom parameters constrained
Δρ_max_ = 1.72 e Å^−3^
Δρ_min_ = −1.15 e Å^−3^

### 

Data collection: *APEX2* (Bruker, 2010 ▶); cell refinement: *SAINT* (Bruker, 2008 ▶); data reduction: *SAINT* and *XPREP* (Bruker, 2008 ▶); program(s) used to solve structure: *SIR97* (Altomare *et al.*, 1999 ▶); program(s) used to refine structure: *SHELXL97* (Sheldrick, 2008 ▶); molecular graphics: *DIAMOND* (Brandenburg & Putz, 2005 ▶); software used to prepare material for publication: *publCIF* (Westrip, 2010 ▶) and *WinGX* (Farrugia, 2012 ▶).

## Supplementary Material

Crystal structure: contains datablock(s) global, I. DOI: 10.1107/S1600536812048465/su2526sup1.cif

Structure factors: contains datablock(s) I. DOI: 10.1107/S1600536812048465/su2526Isup2.hkl

Supplementary material file. DOI: 10.1107/S1600536812048465/su2526Isup3.cdx

Additional supplementary materials:  crystallographic information; 3D view; checkCIF report

## Figures and Tables

**Fig. 1.**
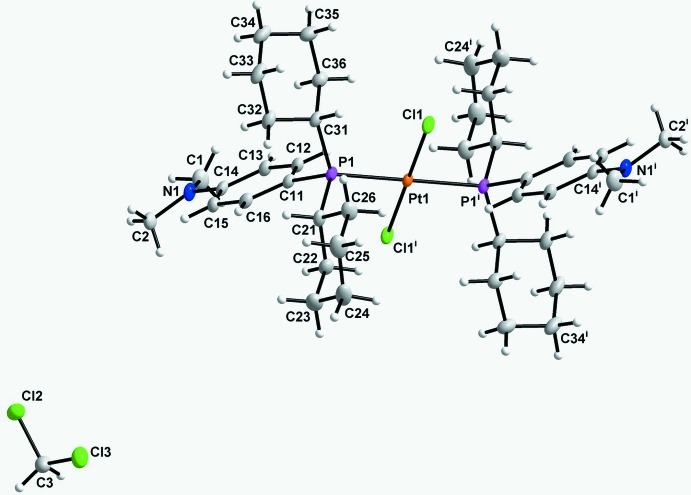
The molecular structure of the title complex, showing the atom numbering and the ordered dichloromethane solvent molecule. The displacement ellipsoids are drawn at the 50% probability level [Symmetry code: (i) -x+3/2, -y+1/2, -z].

## supplementary crystallographic information

### Comment

Transition metal complexes containing phosphine, arsine and stibine ligands are widely being investigated in various fields of organometallic chemistry (Spessard & Miessler, 1996). [PtCl_2_(*L*)_2_] (*L* = tertiary phosphine, arsine or stibine) complexes can conveniently be prepared by the substitution of 1,5-cyclooctadiene (COD) from [PtCl_2_(COD)]. As part of a systematic investigation involving complexes with the general formula *cis/trans*-[*MX*_2_(*L*)_2_] (*M* = Pt or Pd; *X* = halogen, Me, Ph; *L* = Group 15 donor ligand), we have synthesized the title compound and report herein on its crystal structure.

In the title compound, Fig. 1, the Pt atom is located on an inversion center with each pair of equivalent ligands in a mutually *trans* orientation. The geometry is, therefore, square planar and the Pt atom is not elevated out of the coordinating atom plane. All the bond angles in the coordination polyhedron are close to their ideal value of 90°, with P1—Pt1—Cl1 = 90.4 (2)° and P1—Pt1—Cl^i^ = 89.6 (2)°. As required by the crystallographic symmetry, the P1—Pt1—P^i^ and Cl1—Pt1—Cl^i^ angles are both 180° [symmetry code: (i) -*x*+3/2, -*y*+1/2, -*z*.].

To describe the steric demand of the phosphane ligands the Tolman cone angle (Tolman, 1977) is still the most commonly used model. Applying this model to the geometry obtained from the title compound (and adjusting the Pt—P bond distance to 2.28 Å) we calculated an effective cone angle (Otto, 2001) of 164°.

The title compound compares well with other closely related Pt^II^ complexes reported in the literature containing two chloride and two tertiary phosphine ligands in a *trans* geometry (Lutz *et al.*, 2005). The Pt—P and Pt—Cl bond distances of 2.326 (6) and 2.311 (6) Å, respectively for the title compound, fit well into the typical range for complexes of this kind. The title compound crystallizes as a solvated complex which is common for these type of Pt^II^ complexes (Johansson *et al.*, 2002).

The title compound crystallizes with two molecules of dichloromethane. One of these molecules was initially modelled as a severely disordered molecule. We subsequently removed the disordered dichloromethane molecule by applying the SQUEEZE routine as found in *PLATON* (Spek, 2009).

### Experimental

Dichloro(1,5-cyclooctadiene)platinum(II), [PtCl_2_(COD)], was prepared according to the literature procedure (Drew & Doyle, 1990). A solution of dicyclohexyl-[4-(*N,N*-dimethylamino)phenyl]phosphine (63.5 mg, 0.2 mmol) in dichloromethane (2 cm^3^) was added to a solution of [PtCl_2_(COD)] (37.4 mg, 0.1 mmol) in dichloromethane (3 cm^3^). Slow evaporation of the solvent gave colourless crystals of the title compound.

### Refinement

The aromatic, methine, methyl and methylene H atoms were placed in geometrically idealized positions (C—H = 0.95, 1.00, 0.98 and 0.99 Å, respectively) and constrained to ride on their parent atoms, with *U*_iso_(H) = k × *U*_eq_(C) where k = 1.5 for methyl H atoms, and = 1.2 for other H atoms. Methyl torsion angles were refined from electron density.

Large thermal motion of one of the dichloromethane solvate molecules, held only by weak intermolecular hydrogen bonding, is observed. This was initially treated anisotropically as distorted over two partially occupied sites generated by symmetry, with atom C4 restrained isotropically. Different disordered models resulted in unstable refinement cycles. Placement of H atoms on C4 also resulted in unstable refinement. This procedure resulted in unsatisfactory refinements and the molecule was removed by applying the SQUEEZE routine in *PLATON* (Spek, 2009).

### Crystal data

**Table:**

[PtCl_2_(C_20_H_32_NP)_2_]·2CH_2_Cl_2_	*F*(000) = 2176
*M**_r_* = 1070.70	*D*_x_ = 1.445 Mg m^−^^3^
Monoclinic, *C*2/*c*	Cu *K*α radiation, λ = 1.54178 Å
Hall symbol: -C 2yc	Cell parameters from 9651 reflections
*a* = 19.4146 (9) Å	θ = 4.1–65.7°
*b* = 13.1517 (6) Å	µ = 9.16 mm^−^^1^
*c* = 19.3459 (9) Å	*T* = 100 K
β = 94.660 (2)°	Cuboid, colourless
*V* = 4923.4 (4) Å^3^	0.26 × 0.24 × 0.16 mm
*Z* = 4	

### Data collection

**Table:**

Bruker APEX DUO 4K CCD diffractometer	4239 independent reflections
Radiation source: Incoatec IµS microfocus X-ray source	4069 reflections with *I* > 2σ(*I*)
Incoatec Quazar Multilayer Mirror monochromator	*R*_int_ = 0.045
Detector resolution: 8.4 pixels mm^-1^	θ_max_ = 66.2°, θ_min_ = 4.1°
φ and ω scans	*h* = −21→22
Absorption correction: multi-scan (*SADABS*; Bruker, 2008)	*k* = −14→15
*T*_min_ = 0.199, *T*_max_ = 0.322	*l* = −22→21
56178 measured reflections	

### Refinement

**Table:**

Refinement on *F*^2^	Primary atom site location: structure-invariant direct methods
Least-squares matrix: full	Secondary atom site location: difference Fourier map
*R*[*F*^2^ > 2σ(*F*^2^)] = 0.025	Hydrogen site location: inferred from neighbouring sites
*wR*(*F*^2^) = 0.067	H-atom parameters constrained
*S* = 1.08	*w* = 1/[σ^2^(*F*_o_^2^) + (0.0406*P*)^2^ + 10.7442*P*] where *P* = (*F*_o_^2^ + 2*F*_c_^2^)/3
4239 reflections	(Δ/σ)_max_ = 0.001
244 parameters	Δρ_max_ = 1.72 e Å^−^^3^
0 restraints	Δρ_min_ = −1.15 e Å^−^^3^

### Special details

**Table:**

Geometry. All e.s.d.'s (except the e.s.d. in the dihedral angle between two l.s. planes) are estimated using the full covariance matrix. The cell e.s.d.'s are taken into account individually in the estimation of e.s.d.'s in distances, angles and torsion angles; correlations between e.s.d.'s in cell parameters are only used when they are defined by crystal symmetry. An approximate (isotropic) treatment of cell e.s.d.'s is used for estimating e.s.d.'s involving l.s. planes.
Refinement. Highly disordered solvate molecule is observed, resulting in residual electron density around the C4 atom. Different disordered models, however, resulted in unstable refinement cycles. Placement of H atoms on C4 also resulted in unstable refinement. This procedure resulted in unsatisfactory refinements and the molecule was removed by applying the SQUEEZE routine as found in *PLATON* (Spek, 2003).

### Fractional atomic coordinates and isotropic or equivalent isotropic displacement parameters (Å^2^)

**Table:**

	*x*	*y*	*z*	*U*_iso_*/*U*_eq_	
Pt1	0.75	0.25	0	0.01460 (8)	
Cl1	0.85498 (3)	0.19559 (5)	0.05181 (3)	0.02286 (14)	
P1	0.69307 (3)	0.17595 (4)	0.08851 (3)	0.01619 (13)	
C31	0.74703 (13)	0.15612 (19)	0.17086 (12)	0.0202 (5)	
H131	0.7901	0.1206	0.1591	0.024*	
C34	0.78157 (15)	0.1765 (2)	0.32170 (14)	0.0321 (6)	
H13A	0.7403	0.2114	0.337	0.039*	
H13B	0.8142	0.1651	0.363	0.039*	
C36	0.76895 (17)	0.25841 (19)	0.20359 (17)	0.0241 (7)	
H13C	0.7939	0.2985	0.1702	0.029*	
H13D	0.7273	0.2972	0.214	0.029*	
C11	0.61712 (16)	0.24227 (17)	0.11403 (15)	0.0179 (6)	
C15	0.50367 (17)	0.24432 (17)	0.15855 (16)	0.0197 (6)	
H115	0.4654	0.2075	0.1734	0.024*	
C26	0.72650 (14)	−0.0195 (2)	0.04868 (15)	0.0269 (6)	
H12A	0.7522	0.0105	0.0115	0.032*	
H12B	0.758	−0.0229	0.0915	0.032*	
C16	0.56012 (13)	0.19181 (19)	0.13759 (12)	0.0193 (5)	
H116	0.5602	0.1196	0.1393	0.023*	
C13	0.55973 (13)	0.40207 (18)	0.13530 (12)	0.0192 (5)	
H113	0.5605	0.4743	0.1347	0.023*	
C35	0.81549 (19)	0.2434 (2)	0.27030 (18)	0.0302 (8)	
H13E	0.8595	0.2119	0.259	0.036*	
H13F	0.8263	0.3104	0.2918	0.036*	
C25	0.70229 (16)	−0.1267 (2)	0.02768 (17)	0.0352 (7)	
H12C	0.7427	−0.168	0.017	0.042*	
H12D	0.6812	−0.1591	0.067	0.042*	
C24	0.65002 (17)	−0.1253 (2)	−0.03515 (16)	0.0365 (7)	
H12E	0.6335	−0.1954	−0.0453	0.044*	
H12F	0.6726	−0.1003	−0.076	0.044*	
C32	0.71270 (14)	0.0884 (2)	0.22298 (13)	0.0246 (5)	
H13G	0.6687	0.1198	0.2345	0.029*	
H13H	0.702	0.0211	0.2018	0.029*	
C12	0.61541 (13)	0.34844 (19)	0.11366 (12)	0.0188 (5)	
H112	0.6535	0.3848	0.0981	0.023*	
Cl2	0.03909 (4)	0.22618 (7)	0.10388 (4)	0.04094 (18)	
Cl3	0.09563 (4)	0.09162 (6)	0.00234 (4)	0.03576 (17)	
N1	0.44579 (11)	0.40351 (16)	0.17961 (11)	0.0214 (4)	
C14	0.50191 (13)	0.35131 (19)	0.15818 (12)	0.0192 (5)	
C21	0.66396 (13)	0.04799 (19)	0.06127 (13)	0.0204 (5)	
H121	0.6398	0.0172	0.0999	0.025*	
C2	0.38221 (13)	0.3494 (2)	0.18892 (14)	0.0250 (5)	
H2A	0.366	0.3156	0.1455	0.038*	
H2B	0.347	0.3976	0.202	0.038*	
H2C	0.3906	0.2985	0.2256	0.038*	
C3	0.02418 (15)	0.1670 (2)	0.02159 (15)	0.0327 (6)	
H3A	−0.0176	0.1237	0.0212	0.039*	
H3B	0.0157	0.2198	−0.0146	0.039*	
C33	0.76035 (15)	0.0747 (2)	0.28947 (13)	0.0305 (6)	
H13I	0.7362	0.0341	0.3232	0.037*	
H13J	0.8022	0.0367	0.2786	0.037*	
C1	0.44126 (15)	0.5125 (2)	0.17206 (16)	0.0310 (6)	
H1A	0.4849	0.5435	0.1903	0.047*	
H1B	0.4035	0.5382	0.1979	0.047*	
H1C	0.4323	0.5298	0.1229	0.047*	
C22	0.61287 (14)	0.0505 (2)	−0.00345 (13)	0.0258 (6)	
H12G	0.5725	0.093	0.0056	0.031*	
H12H	0.6354	0.0812	−0.0426	0.031*	
C23	0.58883 (16)	−0.0575 (2)	−0.02278 (16)	0.0338 (7)	
H12I	0.5635	−0.0862	0.0151	0.041*	
H12J	0.5568	−0.0552	−0.0653	0.041*	

### Atomic displacement parameters (Å^2^)

**Table:**

	*U*^11^	*U*^22^	*U*^33^	*U*^12^	*U*^13^	*U*^23^
Pt1	0.01470 (11)	0.01799 (11)	0.01167 (10)	0.00316 (4)	0.00452 (6)	0.00001 (4)
Cl1	0.0180 (3)	0.0343 (3)	0.0169 (3)	0.0085 (2)	0.0052 (2)	0.0057 (2)
P1	0.0175 (3)	0.0184 (3)	0.0133 (3)	0.0028 (2)	0.0056 (2)	−0.0004 (2)
C31	0.0228 (12)	0.0243 (12)	0.0139 (11)	0.0056 (10)	0.0048 (9)	0.0003 (10)
C34	0.0322 (15)	0.0480 (17)	0.0161 (13)	0.0121 (13)	0.0018 (11)	−0.0015 (12)
C36	0.0247 (16)	0.0287 (16)	0.0191 (16)	0.0048 (10)	0.0019 (13)	−0.0010 (9)
C11	0.0196 (15)	0.0213 (14)	0.0131 (14)	0.0027 (9)	0.0040 (11)	−0.0005 (8)
C15	0.0199 (15)	0.0240 (15)	0.0159 (15)	0.0004 (9)	0.0051 (12)	0.0021 (8)
C26	0.0276 (14)	0.0200 (13)	0.0343 (15)	0.0039 (11)	0.0102 (11)	−0.0048 (11)
C16	0.0249 (12)	0.0176 (11)	0.0159 (11)	0.0019 (10)	0.0043 (10)	0.0015 (9)
C13	0.0228 (12)	0.0184 (12)	0.0170 (11)	0.0033 (10)	0.0056 (9)	0.0003 (9)
C35	0.0291 (18)	0.0406 (19)	0.0201 (17)	0.0048 (11)	−0.0027 (14)	−0.0042 (10)
C25	0.0375 (16)	0.0223 (14)	0.0480 (18)	0.0024 (12)	0.0176 (14)	−0.0076 (12)
C24	0.0444 (17)	0.0280 (14)	0.0400 (17)	−0.0112 (13)	0.0207 (14)	−0.0143 (13)
C32	0.0291 (14)	0.0271 (13)	0.0186 (12)	0.0059 (11)	0.0082 (11)	0.0029 (10)
C12	0.0207 (12)	0.0213 (12)	0.0147 (11)	−0.0001 (10)	0.0045 (9)	0.0010 (9)
Cl2	0.0401 (4)	0.0589 (4)	0.0238 (4)	−0.0154 (4)	0.0020 (3)	−0.0031 (3)
Cl3	0.0300 (3)	0.0473 (4)	0.0306 (4)	0.0040 (3)	0.0059 (3)	0.0100 (3)
N1	0.0204 (10)	0.0240 (11)	0.0212 (11)	0.0055 (8)	0.0099 (8)	0.0015 (8)
C14	0.0210 (12)	0.0250 (12)	0.0122 (11)	0.0037 (10)	0.0047 (9)	0.0009 (9)
C21	0.0220 (12)	0.0203 (12)	0.0202 (12)	0.0021 (10)	0.0091 (10)	−0.0022 (9)
C2	0.0189 (12)	0.0327 (14)	0.0247 (13)	0.0036 (10)	0.0085 (10)	−0.0007 (11)
C3	0.0257 (14)	0.0487 (18)	0.0236 (14)	−0.0020 (12)	0.0019 (11)	0.0012 (12)
C33	0.0356 (15)	0.0386 (16)	0.0180 (13)	0.0130 (12)	0.0078 (11)	0.0055 (11)
C1	0.0297 (14)	0.0275 (14)	0.0374 (16)	0.0076 (11)	0.0120 (12)	−0.0035 (12)
C22	0.0276 (13)	0.0290 (14)	0.0214 (13)	−0.0013 (11)	0.0057 (11)	−0.0043 (11)
C23	0.0337 (16)	0.0343 (16)	0.0342 (16)	−0.0086 (13)	0.0082 (13)	−0.0110 (13)

### Geometric parameters (Å, º)

**Table:**

Pt1—Cl1^i^	2.3106 (5)	C25—C24	1.519 (5)
Pt1—Cl1	2.3106 (6)	C25—H12C	0.99
Pt1—P1	2.3258 (6)	C25—H12D	0.99
Pt1—P1^i^	2.3258 (6)	C24—C23	1.520 (4)
P1—C11	1.816 (3)	C24—H12E	0.99
P1—C21	1.839 (2)	C24—H12F	0.99
P1—C31	1.853 (2)	C32—C33	1.533 (4)
C31—C36	1.532 (4)	C32—H13G	0.99
C31—C32	1.537 (4)	C32—H13H	0.99
C31—H131	1	C12—H112	0.95
C34—C35	1.517 (5)	Cl2—C3	1.775 (3)
C34—C33	1.520 (4)	Cl3—C3	1.769 (3)
C34—H13A	0.99	N1—C14	1.380 (3)
C34—H13B	0.99	N1—C1	1.443 (4)
C36—C35	1.527 (4)	N1—C2	1.448 (3)
C36—H13C	0.99	C21—C22	1.533 (4)
C36—H13D	0.99	C21—H121	1
C11—C12	1.397 (3)	C2—H2A	0.98
C11—C16	1.398 (4)	C2—H2B	0.98
C15—C16	1.384 (4)	C2—H2C	0.98
C15—C14	1.407 (3)	C3—H3A	0.99
C15—H115	0.95	C3—H3B	0.99
C26—C25	1.530 (4)	C33—H13I	0.99
C26—C21	1.539 (3)	C33—H13J	0.99
C26—H12A	0.99	C1—H1A	0.98
C26—H12B	0.99	C1—H1B	0.98
C16—H116	0.95	C1—H1C	0.98
C13—C12	1.384 (4)	C22—C23	1.532 (4)
C13—C14	1.408 (4)	C22—H12G	0.99
C13—H113	0.95	C22—H12H	0.99
C35—H13E	0.99	C23—H12I	0.99
C35—H13F	0.99	C23—H12J	0.99
			
Cl1^i^—Pt1—Cl1	180.00 (4)	C23—C24—H12E	109.4
Cl1^i^—Pt1—P1	89.64 (2)	C25—C24—H12F	109.4
Cl1—Pt1—P1	90.36 (2)	C23—C24—H12F	109.4
Cl1^i^—Pt1—P1^i^	90.36 (2)	H12E—C24—H12F	108
Cl1—Pt1—P1^i^	89.64 (2)	C33—C32—C31	110.8 (2)
P1—Pt1—P1^i^	180.00 (4)	C33—C32—H13G	109.5
C11—P1—C21	106.27 (11)	C31—C32—H13G	109.5
C11—P1—C31	104.40 (12)	C33—C32—H13H	109.5
C21—P1—C31	104.88 (11)	C31—C32—H13H	109.5
C11—P1—Pt1	116.36 (9)	H13G—C32—H13H	108.1
C21—P1—Pt1	109.01 (8)	C13—C12—C11	121.8 (2)
C31—P1—Pt1	115.00 (8)	C13—C12—H112	119.1
C36—C31—C32	111.0 (2)	C11—C12—H112	119.1
C36—C31—P1	110.51 (18)	C14—N1—C1	120.5 (2)
C32—C31—P1	113.61 (18)	C14—N1—C2	119.7 (2)
C36—C31—H131	107.1	C1—N1—C2	117.1 (2)
C32—C31—H131	107.1	N1—C14—C15	121.0 (2)
P1—C31—H131	107.1	N1—C14—C13	121.9 (2)
C35—C34—C33	111.1 (2)	C15—C14—C13	117.1 (2)
C35—C34—H13A	109.4	C22—C21—C26	110.4 (2)
C33—C34—H13A	109.4	C22—C21—P1	112.19 (18)
C35—C34—H13B	109.4	C26—C21—P1	110.20 (17)
C33—C34—H13B	109.4	C22—C21—H121	108
H13A—C34—H13B	108	C26—C21—H121	108
C35—C36—C31	111.2 (2)	P1—C21—H121	108
C35—C36—H13C	109.4	N1—C2—H2A	109.5
C31—C36—H13C	109.4	N1—C2—H2B	109.5
C35—C36—H13D	109.4	H2A—C2—H2B	109.5
C31—C36—H13D	109.4	N1—C2—H2C	109.5
H13C—C36—H13D	108	H2A—C2—H2C	109.5
C12—C11—C16	117.2 (2)	H2B—C2—H2C	109.5
C12—C11—P1	119.9 (2)	Cl3—C3—Cl2	111.17 (15)
C16—C11—P1	122.85 (17)	Cl3—C3—H3A	109.4
C16—C15—C14	121.1 (3)	Cl2—C3—H3A	109.4
C16—C15—H115	119.4	Cl3—C3—H3B	109.4
C14—C15—H115	119.4	Cl2—C3—H3B	109.4
C25—C26—C21	110.1 (2)	H3A—C3—H3B	108
C25—C26—H12A	109.6	C34—C33—C32	111.4 (2)
C21—C26—H12A	109.6	C34—C33—H13I	109.3
C25—C26—H12B	109.6	C32—C33—H13I	109.3
C21—C26—H12B	109.6	C34—C33—H13J	109.3
H12A—C26—H12B	108.1	C32—C33—H13J	109.3
C15—C16—C11	121.7 (2)	H13I—C33—H13J	108
C15—C16—H116	119.2	N1—C1—H1A	109.5
C11—C16—H116	119.2	N1—C1—H1B	109.5
C12—C13—C14	121.0 (2)	H1A—C1—H1B	109.5
C12—C13—H113	119.5	N1—C1—H1C	109.5
C14—C13—H113	119.5	H1A—C1—H1C	109.5
C34—C35—C36	111.7 (3)	H1B—C1—H1C	109.5
C34—C35—H13E	109.3	C23—C22—C21	110.1 (2)
C36—C35—H13E	109.3	C23—C22—H12G	109.6
C34—C35—H13F	109.3	C21—C22—H12G	109.6
C36—C35—H13F	109.3	C23—C22—H12H	109.6
H13E—C35—H13F	107.9	C21—C22—H12H	109.6
C24—C25—C26	111.9 (2)	H12G—C22—H12H	108.2
C24—C25—H12C	109.2	C24—C23—C22	110.9 (2)
C26—C25—H12C	109.2	C24—C23—H12I	109.5
C24—C25—H12D	109.2	C22—C23—H12I	109.5
C26—C25—H12D	109.2	C24—C23—H12J	109.5
H12C—C25—H12D	107.9	C22—C23—H12J	109.5
C25—C24—C23	111.2 (2)	H12I—C23—H12J	108.1
C25—C24—H12E	109.4		
			
Cl1^i^—Pt1—P1—C11	−34.86 (10)	C36—C31—C32—C33	−55.2 (3)
Cl1—Pt1—P1—C11	145.14 (10)	P1—C31—C32—C33	179.56 (17)
Cl1^i^—Pt1—P1—C21	85.23 (9)	C14—C13—C12—C11	−0.5 (4)
Cl1—Pt1—P1—C21	−94.77 (9)	C16—C11—C12—C13	−0.3 (4)
Cl1^i^—Pt1—P1—C31	−157.36 (9)	P1—C11—C12—C13	−177.42 (19)
Cl1—Pt1—P1—C31	22.64 (9)	C1—N1—C14—C15	−173.3 (3)
C11—P1—C31—C36	−62.6 (2)	C2—N1—C14—C15	−12.9 (4)
C21—P1—C31—C36	−174.20 (19)	C1—N1—C14—C13	6.9 (4)
Pt1—P1—C31—C36	66.1 (2)	C2—N1—C14—C13	167.3 (2)
C11—P1—C31—C32	62.9 (2)	C16—C15—C14—N1	−179.5 (2)
C21—P1—C31—C32	−48.7 (2)	C16—C15—C14—C13	0.3 (4)
Pt1—P1—C31—C32	−168.41 (15)	C12—C13—C14—N1	−179.7 (2)
C32—C31—C36—C35	54.9 (3)	C12—C13—C14—C15	0.5 (4)
P1—C31—C36—C35	−178.1 (2)	C25—C26—C21—C22	−56.8 (3)
C21—P1—C11—C12	−157.2 (2)	C25—C26—C21—P1	178.7 (2)
C31—P1—C11—C12	92.2 (2)	C11—P1—C21—C22	64.7 (2)
Pt1—P1—C11—C12	−35.7 (3)	C31—P1—C21—C22	174.93 (17)
C21—P1—C11—C16	25.8 (3)	Pt1—P1—C21—C22	−61.43 (18)
C31—P1—C11—C16	−84.7 (3)	C11—P1—C21—C26	−171.79 (18)
Pt1—P1—C11—C16	147.4 (2)	C31—P1—C21—C26	−61.6 (2)
C14—C15—C16—C11	−1.2 (4)	Pt1—P1—C21—C26	62.07 (18)
C12—C11—C16—C15	1.2 (4)	C35—C34—C33—C32	−56.0 (3)
P1—C11—C16—C15	178.2 (2)	C31—C32—C33—C34	55.9 (3)
C33—C34—C35—C36	55.7 (3)	C26—C21—C22—C23	58.1 (3)
C31—C36—C35—C34	−55.3 (3)	P1—C21—C22—C23	−178.51 (18)
C21—C26—C25—C24	55.6 (3)	C25—C24—C23—C22	56.3 (3)
C26—C25—C24—C23	−55.5 (3)	C21—C22—C23—C24	−57.8 (3)

Symmetry code: (i) −*x*+3/2, −*y*+1/2, −*z*.
